# Characterization of Voltage-Gated Potassium Channels in Human Neural Progenitor Cells

**DOI:** 10.1371/journal.pone.0006168

**Published:** 2009-07-08

**Authors:** Grit Schaarschmidt, Florian Wegner, Sigrid C. Schwarz, Hartmut Schmidt, Johannes Schwarz

**Affiliations:** 1 Department of Neurology, University of Leipzig, Leipzig, Germany; 2 Carl-Ludwig-Institute for Physiology, University of Leipzig, Leipzig, Germany; 3 Translational Centre of Regenerative Medicine, University of Leipzig, Leipzig, Germany; Universidade Federal do Rio de Janeiro (UFRJ), Instituto de Biofísica da UFRJ, Brazil

## Abstract

**Background:**

Voltage-gated potassium (K_v_) channels are among the earliest ion channels to appear during brain development, suggesting a functional requirement for progenitor cell proliferation and/or differentiation. We tested this hypothesis, using human neural progenitor cells (hNPCs) as a model system.

**Methodology/Principal Findings:**

In proliferating hNPCs a broad spectrum of K_v_ channel subtypes was identified using quantitative real-time PCR with a predominant expression of the A-type channel K_v_4.2. In whole-cell patch-clamp recordings K_v_ currents were separated into a large transient component characteristic for fast-inactivating A-type potassium channels (I_A_) and a small, sustained component produced by delayed-rectifying channels (I_K_). During differentiation the expression of I_A_ as well as A-type channel transcripts dramatically decreased, while I_K_ producing delayed-rectifiers were upregulated. Both K_v_ currents were differentially inhibited by selective neurotoxins like phrixotoxin-1 and α-dendrotoxin as well as by antagonists like 4-aminopyridine, ammoniumchloride, tetraethylammonium chloride and quinidine. In viability and proliferation assays chronic inhibition of the A-type currents severely disturbed the cell cycle and precluded proper hNPC proliferation, while the blockade of delayed-rectifiers by α-dendrotoxin increased proliferation.

**Conclusions/Significance:**

These findings suggest that A-type potassium currents are essential for proper proliferation of immature multipotent hNPCs.

## Introduction

Human neural progenitor cells (hNPCs) isolated from fetal brain tissue are considered a promising source for cell replacement therapies in neurodegenerative disorders [1]. They bear an immense potential to proliferate and represent an appropriate *in vitro* model for investigating mechanisms of early human brain development [2] including ion channel function. The expression of ion channels and their physiological properties are modulated during cell differentiation [3], [4]. Vice versa, ion channels are involved in the regulation of cell differentiation [5]. Proliferation may also be modulated by ion channel activity, whereas the expression of functional voltage-gated potassium (K_v_) channel subtypes seems to be particularly important. For example, proliferation of activated immune cells is repressed by K_v_1.3 blockade [6], and tumor cell divisions are reduced by selective inhibition of Ca^2+^-activated potassium channel subtypes [7]. In contrast, the selective blockade of K_v_1.3 and 3.1 in rat neural progenitor cells increased proliferation [8].

While immature progenitor cells rarely exhibit sodium currents and cannot generate action potentials [9], [10], functional K_v_ channels are expressed early during brain maturation with developmentally regulated and highly cell type specific patterns [11]–[13]. In *Drosophila* CNS precursors, the expression of K_v_ currents seemed to be cell autonomous, while other currents changed, when cell-cell contacts occurred [14]. Therefore, potassium channel function is assumed to be a key requirement for proper progenitor cell proliferation and also may pave the way for neuronal differentiation [15]–[17].

After identification of the four K_v_ channel genes *Shaker*, *Shab*, *ShaI* and *Shaw* in *Drosophila* [18], [19], 8 related gene families were discovered in mammals [20]. Among these, K_v_1, K_v_2, K_v_3 and K_v_4 can form homomeric and heteromeric channels, while K_v_5, K_v_6, K_v_8 and K_v_9 are ‘electrically silent’ and become conducting only after building heteromers with subtypes of K_v_1–4 [21]. Potassium channels regulate neuronal excitability by setting resting membrane potentials as well as firing thresholds and by repolarizing action potentials [22], [23]. In most cells, voltage-activated potassium (K_v_) outward currents exhibit a transient component, which is characterized as the fast-inactivating A-type current (I_A_), and a non-inactivating or slowly inactivating sustained component that comprises delayed-rectifying currents with slow (I_DR_) or fast (I_D_) activation kinetics [24], [25]. Early functional investigations pointed out that I_A_ is typically involved in setting the interspike interval [22], while I_DR_ is essential for fast repolarization of action potentials and consequently contributes to repetitive firing [22], [26]. Biophysical separation of these two currents can be obtained by the design of appropriate voltage protocols [14], [27]. However, due to the diversity of K_v_ channels additional pharmacological isolation of current components is often required [25]. Classical agents to block neuronal K_v_ channels are tetraethylammonium chloride (TEA), which was described to be more effective at blocking I_DR_ [28], and 4-aminopyridine (4-AP), which was commonly used to inhibit I_A_ [29]. Other potent inhibitors of neuronal K^+^ currents are quinidine (QND) a structural isomer of the antidysrhythmic drug quinine, that has been used as a Na^+^ channel blocker [30], and the TEA analogon NH_4_Cl. Naturally occuring toxins like α-dendrotoxin (αDTX), margatoxin (MTX) and phrixotoxin (PTX) are highly selective for single K_v_ channel subtypes and very potent, because of their strong binding affinity [31]–[34].

In the present study we show that proliferating hNPCs express functional K_v_ channels, while they do neither exhibit sodium currents nor action potential firing. An overview of the investigated K_v_1-4 channels and their published functional characteristics is given in Table S1. The expression pattern of K_v_ channel subtypes was investigated in immature hNPCs predominantly expressing the A-type channel transcript K_v_4.2 and in differentiating cells, which showed decreased A-type channel formation. On the other hand, delayed-rectifying channels were upregulated during differentiation. Whole-cell K_v_ currents were separated biophysically into a transient, I_A_-like current and a sustained component denoted as I_K_. Both current components exhibited different sensitivities towards individual K_v_ antagonists, which we utilized to unravel their specific contributions to cell viability and progenitor cell proliferation. The inhibition of I_A_ significantly reduced the proliferation capacity and cell viability, indicating an important role of A-type potassium channels for proliferation and survival of hNPCs.

## Materials and Methods

### Cell culture

Human neural progenitor cells (hNPCs) derived from aborted fetal brain tissue 12 weeks post-fertilization were isolated as described previously [35]–[42]. All tissue procurement was performed according to national guidelines and with approval of local review boards (ethics committee of the University of Leipzig and the “Landesärztekammer Sachsen”). In brief, prior to trituration, the tissue was incubated in 100 µg/ml papain/DNase solution (Roche Diagnostics GmbH, Mannheim, Germany) for 30 min at 37°C, followed by washing with phosphate-buffered saline (PBS), and incubation with antipain (50 µg/ml; Roche) for 30 min at 37°C. Cells were plated on polyornithine and fibronectin (PLO/FN)-precoated culture dishes at a density of 30,000 cells/cm^2^. Expansion of hNPCs was performed in serum-free proliferation medium (PM) based on Dulbecco's modified Eagle medium and Ham's F12 containing the supplements N2 or B27 [2], [42]–[44], the antibiotics Penicillin/Streptomycin (all from PAA Laboratories GmbH, Pasching, Austria), the mitogens epidermal growth factor (EGF) and fibroblast growth factor (FGF2; 20 ng/ml each; both from PAN Biotech GmbH, Aidenbach, Germany). Cells can be stably expanded for prolonged periods (between 10 and 30 passages) in a humidified incubator at 37°C in reduced oxygen (3%) [39], [42], [45]. Differentiation of hNPCs was routinely induced via removal of mitogens and addition of 2% B27, 100 pg/ml Interleukin-1β and 5 µM forskolin (Sigma-Aldrich GmbH, Munich, Germany). This differentiation medium (DM), which was based on Neurobasal medium and additionally contained gentamicin and L-alanyl-L-glutamine (all from GIBCO Invitrogen Corporation, Carlsbad CA, USA), was applied for 2 weeks [10].

### Electrophysiology

Patch pipettes were formed from borosilicate glass (BioMedical Instruments, Zöllnitz, Germany) with a horizontal puller (Sutter Instruments P-97, Novato CA, USA) and fire-polished to final resistances of 2–4 MΩ. The pipette solution contained (mM): 130 KCl, 2 MgCl_2_, 1 CaCl_2_, 10 HEPES, 10 EGTA and 2 Mg-ATP, pH adjusted to 7.3 with KOH (260 mOsm). Poly-L-lysine (PLL)-coated culture dishes (∅ 35 mm) with proliferating hNPCs or differentiated cells were used as recording chamber and filled with a bath solution containing (mM): 150 NaCl, 5.4 KCl, 2 CaCl_2_, 1 MgCl_2_, 10 glucose and 5 HEPES, pH adjusted to 7.3 with NaOH (280 mOsm). Different antagonists (all from Sigma-Aldrich GmbH if not stated otherwise) were dissolved in this bathing solution: 4-aminopyridine (4-AP, 0.1–10 mM), phrixotoxin-1 (PTX, 1–1000 nM, Alomone Labs, Jerusalem, Israel), ammonium chloride (NH_4_Cl, 1–100 mM), quinidine (QND, 0.1–100 µM), α-dendrotoxin (DTX, 1–1000 nM), margatoxin (MTX, 0.1–50 nM) and tetraethylammonium chloride (TEA, 1–100 mM). A fast application system with a triple-barrel glass pipette attached to an electromechanical switching device (SF-77B, Warner Instruments, Hamden, CT, USA) was arranged with the external bath solution flowing centrally and the antagonist solutions flowing through the side tubes. Whole-cell patch clamp experiments were performed at 20–22°C under optical control (inverted microscope DMIL, Leica, Bensheim, Germany). Seal resistances ranged from 1–3 GΩ. Whole-cell currents were amplified using an EPC-9 amplifier (HEKA Elektronik, Lambrecht, Germany), low-pass filtered at 2 kHz, and sampled at 10 kHz. Capacitances were compensated and leak currents were substracted (P/n) using the facilities of the Pulse software (HEKA Elektronik, Lambrecht, Germany). Series resistances (R_s_ = 14±7 MΩ) and liquid junction potentials (V_L_ = 4.3 mV, calculated with Clampex 9.2, Molecular Devices, Sunnyvale, USA) were not corrected.

**Figure 1:**
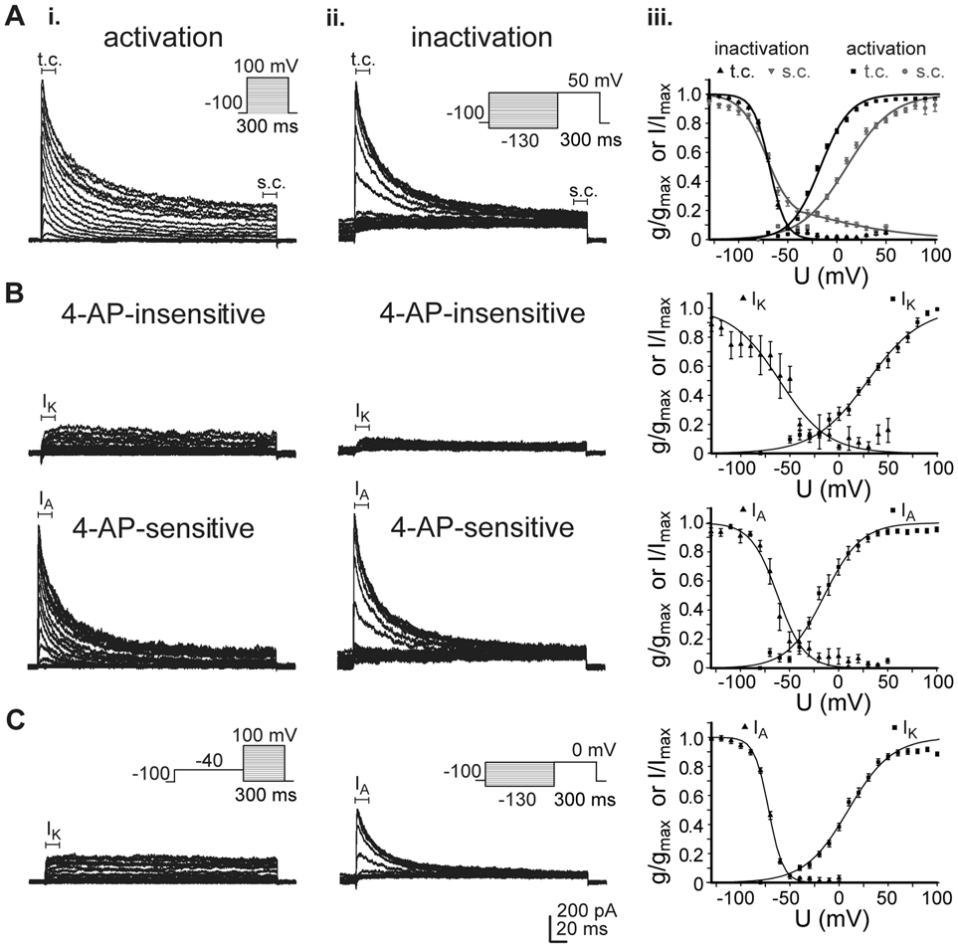
Voltage-activated potassium (K_v_) outward currents in hNPCs. (A): In whole-cell patch-clamp recordings human neural progenitor cells (hNPCs) expressed inactivating A-type (I_A_) and non-inactivating delayed-rectifier-like potassium currents in activation (i) and inactivation protocols (ii, insets). (B): Pharmacological separation of current components was performed by application of 10 mM 4-aminopyridine (4-AP). I_K_ was defined as 4-AP-insensitive component and I_A_ as 4-AP-sensitive component. (C): Biophysical separation of I_K_ was observed in activation protocols by a depolarizing prepulse to −40 mV (500 ms), which caused inactivation of I_A_. In inactivation protocols I_A_ was revealed by a test pulse to 0 mV only since it activated at slightly more negative potentials than I_K_. During each voltage step peak values of the transient component were measured between 0 and 20 ms and sustained currents were determined between 280 and 300 ms. Chord conductances and current values respectively were normalized to their peak amplitudes and fitted to a Boltzmann distribution and current-voltage-relationships of control currents (A), pharmacologically (B) as well as biophysically (C) separated currents were calculated (iii, see Tab. 1). Note the similar I–V relations for both separation procedures.

Voltage-gated currents were activated from a holding potential of −100 mV by depolarizing steps to 100 mV in 10 mV increments (300 ms). Steady-state inactivation of K_v_ currents was determined via hyperpolarizing prepulses increasing in 10 mV increments from −130 mV to 50 mV (500 ms) followed by a test pulse to 50 mV (300 ms). Current amplitudes were measured between 0 and 20 (transient component, t.c.) and between 280 and 300 ms (sustained component, s.c.) of each depolarizing voltage pulse. Biophysical separation of a delayed-rectifier current (I_K_) was obtained in activation protocols by a depolarizing prepulse to −40 mV (500 ms), which inactivated the transient A-type current (I_A_). I_A_ could be isolated in inactivation protocols by a test pulse to 0 mV, because it activated at slightly more negative potentials than I_K_. Both current components were additionally separated pharmacologically by application of 10 mM 4-AP to proliferating hNPCs, with I_K_ being identified as the 4-AP-insensitive component measured in activation protocols and I_A_ was isolated by subtracting the 4-AP-insensitive component of steady-state inactivation currents from control currents (Fig. 1). K_v_ currents evoked in activation protocols were converted to chord conductances assuming a reversal potential (V_rev_) of −82 mV (calculated according to 130 mM K^+^ inside/5.4 mM K^+^ outside). Values were normalized to the peak amplitudes and fitted to the Boltzmann distribution using Origin 6.1 (OriginLab Corporation, Northampton MA, USA):
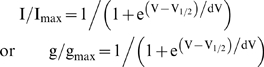
(1)where V_1/2_ is the half maximal activation/inactivation, and dV the slope of the voltage dependency.

For dose-response relationships the inhibition of biophysically separated peak currents was determined during a single depolarizing voltage step from −100 mV to 100 mV (−40 mV prepulse, for I_K_) or to 0 mV (−130 mV prepulse, for I_A_). At the same time antagonists were applied starting 30 s prior to the test pulses. Values were normalized to peak amplitudes recorded in the absence of antagonists and fitted with the Hill equation using Origin 6.1:
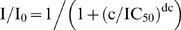
(2)with IC_50_ being the half maximal inhibitory concentration, and dc the Hill coefficient determining the slope of the concentration dependency.

### Total RNA isolation and PCR analysis

Total RNA was isolated from proliferating hNPCs as well as from differentiated cells (4 tissue preparations each) grown in 75 cm^2^ PLO/FN-precoated culture flasks using the RNeasy mini kit (QIAGEN Sciences, Germantown MD, USA) according to the manufacturer's protocol. First-strand cDNA was prepared from total RNA using the RevertAid first strand cDNA synthesis kit (Fermentas International Inc., Burlington, Canada). 30 µl samples of total RNA were transcribed to cDNA with 600 U of reverse transcriptase. The reaction mixture of 60 µl further contained 5 µM oligo(dT)_18_ primer, 0.5 mM nucleotide triphosphates (dNTPs), 50 mM KCl, 4 mM MgCl_2_, 10 mM dithiothreitol (DTT) and 50 mM Tris-HCl (pH 8.3). Oligonucleotide primers for subtypes of the K_v_ channel families 1–4 (see Table S2; MWG Biotech AG, Ebersberg, Germany) were designed to flank intron sequences, if feasible, using Primer 3 software (http://frodo.wi.mit.edu) and tested by means of conventional PCR analysis. PCR samples contained: 100 ng cDNA, 0.625 U Taq DNA polymerase (Fermentas International Inc., Burlington, Canada), 2 µM forward and reverse primers, 1 mM dNTPs, 50 mM KCl, 2.5 mM MgCl_2_ and 10 mM Tris-HCl (pH 8.8) in a final volume of 25 µl. The amplifications were performed in a Peltier thermal cycler (MJ Research Inc., Bio-Rad, Watertown MA, USA) using the following protocol: 95°C for 4 min to activate the Taq polymerase, followed by 30 cycles of 95°C for 45 s, 55°C for 40 s and 72°C for 1 min, amplification was stopped at 72°C for 10 min. Aliquots of the PCR reactions were analysed by 2% agarose gel electrophoresis and visualized by ethidium bromide fluorescence using a MultiImage light cabinet and the analysis software AlphaImager 120 v. 5.1 (Alpha Innotech Corporation, San Leandro CA, USA).

Quantitative real-time PCR was performed using 300 ng cDNA from total RNA, 600 nM forward and reverse primers, Platinum-SYBR Green qPCR Supermix® (SYBR Green I, 0.375 U Platinum Taq DNA polymerase, 20 mM Tris-HCl (pH 8.4), 50 mM KCl, 3 mM MgCl2, dNTPs 200 µM each, 0.25 U UDG) and 100 nM 6-carboxy-X-rhodamine (both from Invitrogen) using the following protocol in an MX 3000P instrument (Stratagene, La Jolla, CA, USA): 2 min 50°C, 2 min 95°C and 50 cycles of 15 s 95°C, 30 s 60°C. To confirm a single amplicon a product melting curve was recorded. Threshold cycle (Ct) values were placed within the exponential phase of the PCR as described previously by Engemaier et al. (2006). Ct values of 4–12 independent experiments, each performed in duplicate, were normalized to ribosomal protein L22 (Ct−Ct RPL22 = ΔCt) [46]. ΔCt values were converted to 2^−^Δ^Ct^ to calculate the relative expression levels [35].

### Cell viability

Evaluation of cell viability was performed by a tetrazolium salt assay using the reagent 3-(4,5-Dimethylthiazol-2-yl)-2,5-diphenyltetrazolium bromide (MTT, Sigma-Aldrich GmbH). In viable cells MTT is converted by the mitochondrial dehydrogenase to a blue formazan product [47], [48], [49]. HNPCs were seeded into 96-well PLO/FN-precoated culture plates (10,000 cells/well, 3 tissue preparations) and incubated for 24 h at 37°C before K_v_ antagonists were added. Cells were treated for 72 h with different concentrations of 4-AP (0.1–2 mM), PTX (1–1000 nM), NH_4_Cl (1–100 mM), QND (5–100 µM), DTX (0.01–10 µM), MTX (1–500 nM) and TEA (1–100 mM). Additionally, electrophysiologically determined inhibitory doses (IC_50_/IC_80_) were used to compare the effects on cell viability (for each concentration n≥4 well). Untreated cells were used as control. After the culture period, 10 µl of 5 mg/ml MTT stock solution were added to each well. Following additional 4 h of incubation at 37°C, culture medium was rejected. To solubilize the formazan crystals 100 µl of 5% acid isopropyl alcohol was applied to the adherent cells and plates were placed on a shaker for at least 30 s. Cell viability was determined colorimetrically at 570 nm using the automated Synergy HT multi-mode microplate reader equipped with the analysis software Gen 5 (BioTek Instruments Inc., Winooski, VT, USA). Absorbance values were normalized to control values of untreated cells.

According to this, a flow cytometric analysis was performed to substantiate the effects on cell cycle (see Methods S1).

### Cell proliferation

Progenitor cell proliferation was quantified by a colorimetric immunoassay based on the measurement of 5-bromo-2-deoxyuridine (BrdU) incorporation during DNA synthesis [50] (cell proliferation ELISA, Roche Diagnostics GmbH, Mannheim, Germany). HNPCs were seeded into 96-well PLO/FN-precoated culture plates (10,000 cells/well, 3 tissue preparations) and incubated for 24 h at 37°C before K_v_ antagonists were added. Cells were treated for 72 h with electrophysiologically determined inhibitory doses (IC_50_/IC_80_) of 4-AP, PTX, NH_4_Cl, QND, DTX, MTX and TEA (for each concentration n≥4 well). Untreated cells were used as control. After the culture period, 100 µM BrdU was added to each well. During a labeling period of 4 h at 37°C the pyrimidine analogue BrdU was incorporated in place of thymidine into the DNA of proliferating cells. After rejecting the labeling medium 200 µl FixDenat solution were added to each well to fix the cells and denature DNA during an incubation period of 30 min at 20–22°C. The fixing solution was rejected and 100 µl/well anti-BrdU-POD antibody solution was added to bind the incorporated BrdU. The cells were incubated for 90 min at 20–22°C and subsequently washed 3 times with phosphate-buffered saline. By adding 100 µl/well tetramethylbenzidine solution the substrate reaction was started and immune complexes were detected within 5–10 min. The reaction product was quantified by measuring the absorbance at 370 nm (reference wavelength 492 nm) using a scanning multiwell spectrophotometer equipped with the analysis software Gen 5 (Synergy HT multi-mode microplate reader, BioTek Instruments Inc., Winooski, VT, USA). The absorbance values directly correlate to the amount of DNA synthesis and hereby to the number of proliferating cells and were normalized to absorbance values of untreated cells.

### Statistical analysis

Data were expressed as mean±standard error (SEM). Statistical differences were calculated with Students's t-test (two-tailed, unpaired) using Origin 6.1 (OriginLab Corporation, Northampton MA, USA) or one-way ANOVA, followed by Tukey's post-hoc test using GraphPad Prim 3 (GraphPad Software Inc., La Jolla, USA); p values≤0.05 were considered significant.

## Results

### Voltage-gated potassium currents in proliferating hNPCs

To characterize the voltage-dependency of voltage-gated potassium (K_v_) currents in proliferating human neural progenitor cells (hNPCs) outward currents were elicited in whole-cell voltage-clamp recordings either in activation protocols or steady-state inactivation protocols (Fig. 1A). We found that outward currents were composed of a transient (t.c.) and a sustained component (s.c.). Typically, the inactivating current component is considered as I_A_ and the sustained component as I_DR_ or I_D_ [22], [24]. We did not distinguish between I_DR_ and I_D_ and denoted the sustained component as I_K_. The transient component of whole-cell potassium outward currents reached maximal capacitance-corrected current densities of 329±42 pA/pF in activation protocols, while the sustained component measured only 56±7 pA/pF (n = 35–38). In inactivation protocols lower current densities were obtained (t.c. 227±23 pA/pF, s.c. 21±4 pA/pF, n = 36–38; Fig. 1A) due to the reduced driving force. Inactivation data of the sustained current (s.c.) were best fit with a sum of two Boltzmann equations (Fig. 1A iii). Because the first component showed values similar to the transient component, this likely reflects the contribution of I_A_ to the sustained component.

I_A_ and I_K_ were pharmacologically separated by application of 10 mM 4-aminopyridine (4-AP). I_K_ was classified as 4-AP-insensitive current in activation protocols (30±5 pA/pF, n = 10–13) and contributed 10% to the transient and 47% to the sustained whole-cell current. I_A_ was isolated as 4-AP-sensitive component during steady-state inactivation (207±66 pA/pF, n = 6–7) and constituted 90% of the transient and 53% of the sustained component of K_v_ outward currents (Fig. 1B). In addition, biophysical separation of I_K_ was performed in activation protocols by a depolarizing prepulse to −40 mV, which caused inactivation of I_A_. The biophysically measured I_K_ amplitudes (32±3 pA/pF, n = 36–38) were comparable to pharmacologically determined values. Also the voltage dependency was similar, while half-maximal activation values differed between the two separation methods. Because I_A_ was activated at slightly more negative potentials than I_K_, it was isolated in inactivation protocols by a test pulse to 0 mV and had amplitudes of 96±8 pA/pF (n = 33–36) - smaller than the pharmacologically separated I_A_, which we attribute to the smaller driving force during depolarization to 0 mV instead of 50 mV. The current-voltage relationships of I_A_ were similar with pharmacological and biophysical separation (Fig. 1C, Tab. 1), indicating that the same current was separated. In the following experiments biophysical separation was used, since we determined the sensibility of I_A_ and I_K_ against different K_v_ antagonists in dose-response curves.

**Table 1:** Voltage dependency of K_v_ currents.

Protocol	activation						inactivation					
	t.c./I_A_			s.c./I_K_			t.c./I_A_			s.c./I_K_		
Parameter	V_1/2_ (mV)	dV (mV/e)	n	V_1/2_ (mV)	dV (mV/e)	n	V_1/2_ (mV)	dV (mV/e)	n	V_1/2_ (mV)	dV (mV/e)	n
**PM**												
control	−16.7±1.1	15.0±1.0	36–38	10.0±3.6	8.2±0.5	22–36	−68.6±0.6	23.2±3.1	38	−33.1±3.9	52.7±5.2	14–35
pharma-cological separation	−14.5±1.6	18.8±1.5	4–13	29.2±1.5	27.4±1.5	9–10	−62.1±1.7	13.6±1.5	5–7	−60.6±2.7	24.5±2.7	3–7
biophysical separation				9.4±1.5	22.1±1.4	21–37	−71.7±0.5	7.3±0.4	36			
**DM**												
control	−15.5±1.9	16.6±1.7	26–27	−0.6±1.3	12.9±1.2	20–27	−73.2±1.4	7.9±1.0	27	−4.9±2.4	12.0±1.9	9–27
biophysical separation				5.7±1.4	11.3±1.2	5–25	−76.7±0.9	7.8±0.8	13–22			

Parameters of I–V curves fitted to the Boltzmann distribution with V_1/2_ being the half maximal activation/inactivation, and dV the slope of the voltage dependency. Control inactivation data of the sustained current (s.c.) best fit with a sum of two Boltzmann equations, and because the first component had values similar to the transient current (t.c., I_A_), only the values of the more depolarized component, assumed to represent I_K_, are shown. All data presented as mean±SD.

In proliferating hNPCs half-maximal activation of I_K_ was determined at 10 to 30 mV by fitting activation curves of normalized chord conductances to the Boltzmann distribution. Fitted inactivation curves of current values showed half-maximal inactivation of I_A_ at −60 to −70 mV (Fig. 1iii, Table 1). Whole-cell K_v_ currents were constituted to 90% by I_A_ and to 10% by I_K_. Thus, A-type currents are the predominant potassium outward currents in immature, proliferating hNPCs.

### Comparison of K_v_ currents in hNPCs and differentiated cells

**Figure 2:**
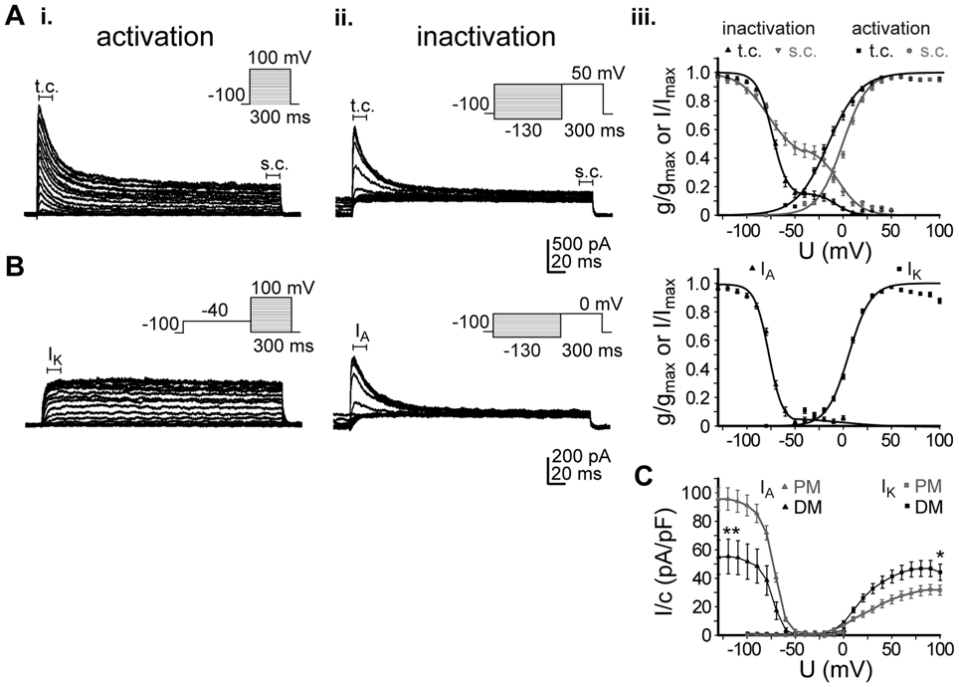
Voltage-activated potassium (K_v_) currents in differentiated cells. Potassium outward currents evoked in hNPCs, which were differentiated for 14 days in differentiation medium (DM). (A): Transient (t.c.) and sustained (s.c.) whole-cell K_v_ currents elicited via activation (i) and inactivation protocol (ii, insets) were measured between 0 and 20 ms and between 280 and 300 ms, respectively, of each depolarizing voltage pulse. Chord conductances and current values were normalized to their peak amplitudes and fitted to a Boltzmann distribution (iii, see Tab. 1). Inactivation data of the sustained current were best described by a sum of two Boltzmann equations. Thereby the second component seemed to increase during differentiation. (B): I_K_ (i) and I_A_ (ii) were biophysically separated in activation or inactivation protocols as described in Fig. 1. (C): Current values of I_K_ and I_A_ normalized to cell capacitances for cells grown in proliferation medium (PM) and in differentiation medium (DM). I_K_ significantly increased, while I_A_ decreased in differentiated cells compared to hNPCs (unpaired t-test, *p<0.05, **p<0.01).

To investigate the development of K_v_ currents during differentiation, hNPCs were exposed to a differentiation medium (DM) for 14 days prior to the recording (Fig. 2A, B). Differentiated hNPCs represent a heterogenous population of cells composed of neurons (∼50% Tuj1 positive), astrocytes (∼30% GFAP-positive), oligodendrocytes and cells that do not differentiate [10]. After 14 days of differentiation they exhibited no remarkable expression of sodium inward currents, which is consistent with Schaarschmidt et al. (2009) [10].

The biophysically separated I_K_ showed similar half-maximal activation (6 mV in DM vs. 91 mV in PM), but lower voltage dependency (11 mV/e-fold in DM vs. 22 mV/efold in PM). Current-voltage relationships of the transient I_A_ were comparable - half-maximal inactivation at −72 mV in DM vs. −77 mV in PM, voltage dependency 8 mV/e-fold in DM vs. 7 mV/e-fold in PM (Fig. 2iii, Tab. 2).

**Table 2:** Concentration dependency of inhibited K_v_ currents.

Protocol	activation				inactivation			
	I_K_				I_A_			
Parameter	IC_50_	IC_80_	dc	n	IC_50_	IC_80_	dc	n
Inhibitor								
**4-AP** (mM)	0.5±0.1	-	2.5±0.1	3–9	1.7±0.3	4.6	1.4±0.2	7–9
**PTX** (µM)	-	-	-	-	1.8±0.7	28.4	0.5±0.1	3–8
**NH_4_Cl** (mM)	255.6±7.7	811.5	1.2±0.1	6–8	35.5±2.4	159.6	0.9±0.1	6–8
**QND** (µM)	3.4±0.3	18.3	0.8±0.1	8–9	42.0±10.5	531.4	0.5±0.1	9
**DTX** (nM)	163.9±20.9	2622.1	0.7±0.1	14	-	-	-	-
**TEA** (mM)	18.4±5.9	293.9	0.5±0.1	6–8	48.7±6.0	164.0	1.1±0.2	8

Parameters of dose-response relationships fitted with the Hill equation, where IC_50_ is the half maximal, IC_80_ the 80 percent inhibitory concentration and dc the Hill coefficient determining the slope of the concentration dependency. All data presented as mean±SD.

Furthermore, in differentiated cells the mean current density of I_K_ was significantly increased (45±6 pA/pF in DM vs. 29±3 pA/pF in PM, n = 23–36), while I_A_ amplitudes decreased (54±12 pA/pF vs. 96±8 pA/pF in PM, n = 22–36; Fig. 2C). Thus, during differentiation I_K_ seems to be upregulated, while I_A_ is smaller than in proliferating hNPCs.

### Biological equivalents of voltage-gated potassium currents (K_v_)

**Figure 3:**
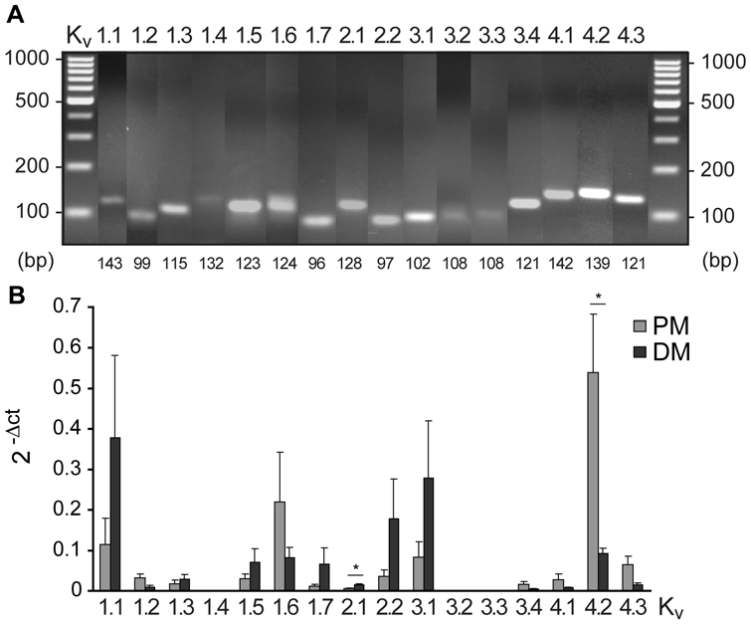
Expression of voltage-gated potassium (K_v_) channels in hNPCs. (A): Identification of K_v_ channels was performed via reverse transcription PCR analysis in proliferating hNPCs (PM) as well as in differentiated cells (DM) after isolation of total mRNA using specific primers for K_v_1–4 subtypes given in Tab. S1 (product sizes below the image). DNA ladders reached from 100–1000 base pairs (bp). (B): For quantification real-time PCR analysis was performed for each K_v_ channel transcript. Threshold cycle (Ct) values were normalized to the Ct values of the house keeping gene and are given as 2^−^Δ^Ct^ (ΔCt = Ct−Ct ribosomal protein L22 (RPL22)). Note the predominant expression of the A-type K_v_4.2 channel transcript in proliferating hNPCs, which was decreased in DM, while the delayed-rectifier channel transcripts K_v_1.1, 1.7, 2.1, 2.2 and 3.1 increased (n≥2, 4 tissue preparations; unpaired t-test, *p<0.05).

K_v_ channel subtypes in proliferating hNPCs as well as in differentiated cells were identified using reverse transcription polymerase chain reaction (RT-PCR) analysis based on mRNA expression. Specific primers for several K_v_ channel subtypes were designed and tested by means of conventional PCR (see Table S2, Fig. 3A). A comprehensive expression pattern of almost all tested subtypes of the K_v_ channel families 1–4 was detected in hNPCs except K_v_1.4, 3.2 and 3.3). This broad spectrum of K_v_ channels was maintained during differentiation.

The expression of several K_v_ channel transcripts was quantified by real-time PCR analysis (Fig. 3B). The A-type channel transcript K_v_4.2 showed the highest expression level and, thus, seemed to contribute predominantly to the generation of K_v_ currents in proliferating hNPCs. During differentiation the expression of the A-type channel K_v_4.2 was significantly downregulated. Also the expression of other A-type channels decreased, while the delayed-rectifier transcripts K_v_1.1, 1.7, 2.1, 2.2 and 3.1 considerably increased. This is in line with the electrophysiologically observed increase in I_K_ and decrease in I_A_ in differentiated cells compared to immature hNPCs.

### Pharmacological inhibition of K_v_ currents in hNPCs

**Figure 4:**
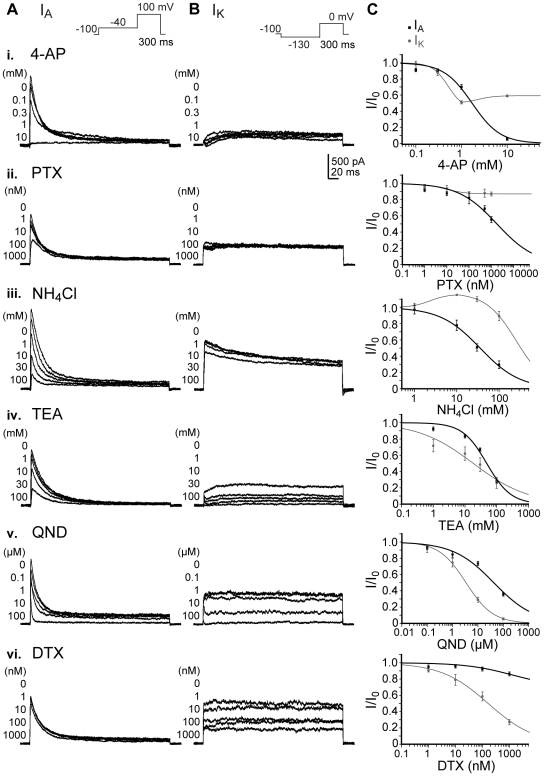
Pharmacological inhibition of K_v_ currents in hNPCs. Biophysically separated A-type (I_A_) and delayed-rectifying (I_K_) K_v_ currents in proliferating hNPCs were differentially inhibited by the 4-aminopyridine (4-AP, i), phrixotoxin-1 (PTX, ii), ammonium chloride (NH_4_Cl, iii), tetraethylammonium chloride (TEA, iv), quinidine (QND, v) and α-dendrotoxin (DTX, vi). (A): Peak amplitudes of I_A_ were measured during a depolarizing voltage step from −130 mV to 0 mV between 0 and 20 ms (inset). (B): I_K_ was determined between 280 and 300 ms of a 100 mV depolarization step following a −40 mV prepulse during the application of different antagonist concentrations (insets). (C): Both current values were normalized to the non-inhibited peak amplitudes. Dose-response relationships were fitted with the Hill equation and IC_50_ values were determined (see Tab. 2). Note that PTX selectively and 4-AP preferentially inhibited I_A_, while DTX selectively blocked I_K_.

There is a broad spectrum of specific and less specific K_v_ antagonists [25]. To selectively inhibit either I_A_ or I_K_ we tested some of the most frequently used blockers on hNPCs and monitored the concentration-dependency of their inhibitory effects on the biophysically separated current components (Fig. 4, Table 2). We started with 4-aminopyridine (4-AP) typically considered as K_v_ blocker preferentially affecting I_A_, but with moderate specificity [24], [29]. 4-AP inhibited I_A_ with IC_50_ = 1.7 mM and a Hill slope of 1.4 (Fig. 4i). I_K_ was not completely blocked. To selectively inhibit I_A_ the spider toxin phrixotoxin-1 was used, which acts as an antagonist on K_v_4.2 and 4.3 channels [34]. Because in hNPCs I_A_ is predominantly carried by K_v_4.2 (Fig. 3B, Tab. S1), this current component was sufficiently blocked with IC_50_ = 1.8 µM and slope 0.5, while I_K_ was not affected (Fig. 4ii). The quaternary ammonium salt NH_4_Cl was actually used to inhibit I_K_. Because of the higher polarity compared to its analogon tetraethylammonium chloride (TEA), it is considered to act on the outer quaternary ammonium ion receptor of K_v_ channels [51]. Surprisingly, it stronger inhibited I_A_ (IC_50_ = 35.5 µM, slope 0.9) than I_K_ (IC_50_ = 255.6 µM, slope 1.2; Fig. 4iii). But compared to 4-AP and PTX high doses were required.

Furthermore, the classical potassium channel antagonist TEA, which is typically used to block I_K_, but with moderate specificity, was applied to the cells [24], [28]. TEA blocked I_K_ with an IC_50_ value of 18 mM and a Hill slope of 0.5 marginally stronger than I_A_ with IC_50_ = 49 mM and slope 1.1 (Fig. 4iv). As a fifth antagonist quinidine (QND) - a classical Na^+^ channel blocker, which is reported to non-specifically block I_K_ and I_A_ - was tested [30], [52]. We found that in hNPCs the IC_50_ value for I_K_ inhibition (IC_50_ = 3.4 µM, slope 0.8) was significantly lower than for blocking I_A_ (IC_50_ = 42.0 µM, slope 0.8; Fig. 4v). Additionally, the neurotoxins α-dendrotoxin (DTX) and margatoxin respectively (MTX; see Results S1) were applied, which are considered to specifically affect K_v_1 subtypes [31]. We found that both selectively blocked I_K_ (DTX with IC_50_ = 163.9 nM and Hill slope 0.7, MTX with IC_50_ = 180.7 nM and slope 0.5), while they were ineffective in blocking I_A_ (Fig. 4v, Fig. S1). Because the channel transcripts K_v_1.2 and 1.3 showed low expression levels, MTX predominantly inhibited K_v_1.1, while DTX additionally blocked K_v_1.6 (Fig. 3B, Tab. S1).

Taken together, 4-AP and NH_4_Cl preferentially and PTX specifically blocked I_A_, while QND stronger and DTX selectively inhibited I_K_. TEA acted as a non-specific K_v_ channel blocker in hNPCs.

### Biological effects of K_v_ channel inhibition in hNPCs

**Figure 5:**
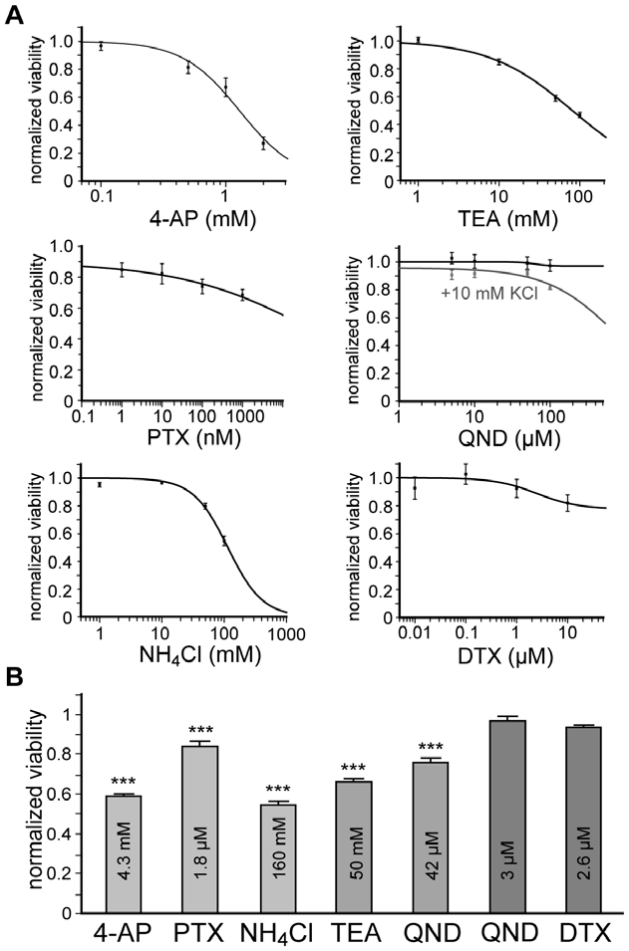
Cell viability after inhibition of voltage-gated potassium (K_v_) channels. Determination of cell viability in proliferating hNPCs via 3-(4,5-Dimethylthiazol-2-yl)-2,5-diphenyltetrazolium salt (MTT) assay. (A): Cell viability was measured colorimetrically after 72 h of K_v_ channel inhibition with different concentrations of 4-aminopyridine (4-AP), phrixotoxin-1 (PTX), ammonium chloride (NH_4_Cl), tetraethylammonium chloride (TEA), quinidine (QND) and α-dendrotoxin (DTX) and normalized to control values without addition of inhibitor. (B): Viability of hNPCs was significantly reduced by electrophysiologically determined inhibitory doses (IC_50_/IC_80_) of 4-AP, PTX and NH_4_Cl, which specifically blocked I_A_, as well as by TEA and higher doses of QND, which inhibited both current components (n≥4, 3 tissue preparations; one-way ANOVA, followed by Tukey's post-hoc test, ***p<0.001).

We further investigated whether K_v_ channels play a role in cell survival. Towards this end, we applied various concentrations of the K_v_ antagonists for 3 days prior to analysis by MTT (3-(4,5-Dimethylthiazol-2-yl)-2,5-diphenyltetrazolium bromide) assay, which colorimetrically measured the production of MTT formazan in viable cells (Fig. 5A). According to the above findings, 4-AP, PTX and NH_4_Cl were utilized to inhibit I_A_, while QND (low doses) and DTX were applied as specific I_K_ blockers. TEA and higher doses of QND blocked all K_v_ currents. For QND treatment the extracellular KCl concentration had to be raised to 10 mM in order to achieve sufficient inhibitory effects, probably due to its action as an open channel blocker [53], [54].

A significant reduction of cell viability was observed after blocking I_A_ with electrophysiologically determined inhibitory doses (IC_50_/IC_80_) of 4-AP, PTX and NH_4_Cl as well as after treatment with TEA and QND, which blocked all K_v_ currents. On the contrary, low doses of QND and DTX, which specifically inhibited I_K_, did not affect cell viability (Fig. 5B). To substantiate these results, cell cycle analysis was performed, that yielded a similar increase in apoptosis after inhibition of I_A_ and all K_v_ currents respectively, while specific I_K_ antagonists did not induce cell death (see Results S2, Fig. S2).

**Figure 6:**
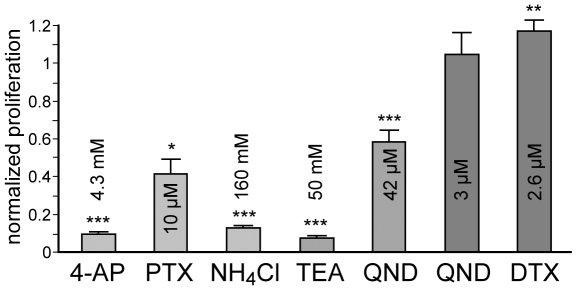
Influences *of voltage-gated potassium (K_v_) channel inhibition* on progenitor c*ell proliferation*. Proliferation of hNPCs was analyzed via BrdU incorporation assay. (A): Progenitor cell proliferation was measured colorimetrically after 72 h of K_v_ channel inhibition and normalized to control values without addition of inhibitor. Electrophysiologically determined inhibitory doses (IC_50_/IC_80_) of 4-aminopyridine (4-AP), phrixotoxin-1 (PTX), ammonium chloride (NH_4_Cl), tetraethylammonium chloride (TEA), quinidine (QND) and α-dendrotoxin (DTX) were applied. Progenitor cell proliferation was significantly reduced by inhibition of I_A_ with 4-AP, PTX, NH_4_Cl as well as by unspecific blockers like TEA and higher doses of QND. In contrast, the I_K_ antagonist DTX increased proliferation of hNPCs (n≥4, 3 tissue preparations; one-way ANOVA, followed by Tukey's post-hoc test, *p<0.05, **p<0.01, ***p<0.001).

To unravel the contribution of K_v_ channels to progenitor cell proliferation we performed a BrdU assay after 3 days of potassium channel blockade according to MTT assay. The inhibition of A-type K_v_ channels by 4-AP, PTX and NH_4_Cl significantly impaired cell proliferation. Blocking all K_v_ currents by TEA and QND had similar effects. In contrast, specific inhibition of delayed-rectifier channels by low doses of QND did not affect proliferation, while the application of DTX even increased proliferation of hNPCs (Fig. 6).

Taken together, these results demonstrate a substantial effect of K_v_ currents on cell survival and proliferation, mainly mediated by I_A_.

## Discussion

We tested the hypothesis that voltage-gated potassium (K_v_) channels play a functional role in the development of human neural progenitor cells (hNPCs).

In whole-cell patch clamp recordings the biophysical separation of two K_v_ currents, I_A_ and I_K_, was obtained by different voltage protocols. The transient current I_A_ was determined in steady-state inactivation protocols by a test pulse to 0 mV, because it is activated at slightly more negative potentials than I_K_. I_K_ was measured as the sustained outward current in activation protocols following a prepulse to −40 mV, which inactivated I_A_. Two types of delayed rectifier currents have been described previously: I_DR_ and I_D_. While I_DR_ is slowly activated with a time to peak of 50–100 ms and does not show pronounced steady-state inactivation, the delay current I_D_ is rapidly activated and slowly inactivated [24], [25]. We could not distinguish between I_DR_ and I_D_ and denoted this current as I_K_. Similar currents for I_A_ and I_K_ were obtained by pharmacological separation. Whole-cell K_v_ currents were constituted to 90% by I_A_ and to 10% by I_K_.

During differentiation I_K_ amplitudes increased, while I_A_ decreased without considerable changes in current-voltage dependencies. An increase in voltage-activated K_v_ currents during development was observed before in several other cell types, for example in rat retinal ganglion cells [55] or in rat cerebellar granule cells [56]. However, downregulation of I_A_ has not been described so far.

In hNPCs a broad pattern of K_v_ channel subtypes was detected with almost all K_v_1–4 channels being expressed except K_v_1.4, 3.2 and 3.3. The A-type channel transcript K_v_4.2 showed predominant expression levels and, thus, seems to have a critical impact on the physiological characteristics of immature progenitor cells. However, expression of K_v_ channel mRNA and electrophysiological or pharmacological K_v_ properties are quite distinct [25]. Although the *in vitro* expression of individual α subunits lead to generation of either classical I_A_ or I_DR_ currents [57], [58], the physiological properties may be dramatically changed by formation of heteromultimers [59], β subunit association [60], [61], the degree of phosphorylation [62], [63] as well as the oxidative state [64], [65]. Therefore, we combined molecular expression studies with the physiological and pharmacological characterization of K_v_ channels. Whereas the high expression of K_v_4.2 mRNA is in line with the 90 percent contribution of I_A_ to whole-cell K_v_ currents, I_K_-producing delayed-rectifier channels are less prominent. Recently, in rat NPCs derived from the subventricular zone I_A_ was found to be mediated by K_v_4.3 and I_K_ by K_v_2.1 [9], while in rat midbrain-derived NPCs high levels of the DR channels K_v_1.3 and K_v_3.1 as well as the A-type channel K_v_1.4 were expressed [8]. Thus, K_v_ channel expression seems to be not only region, but also species specific. During differentiation of hNPCs the formation of A-type channels significantly decreased, while delayed-rectifying channels are upregulated analogous to a reduction in I_A_ and an increase in the generation of I_K_ currents.

Pharmacological investigations revealed different sensitivities of I_A_ and I_K_ to the applied K_v_ antagonists. PTX selectively blocked K_v_4.2 and 4.3 [34], which contribute largely to I_A_, and, thus was sufficient in blocking A-type currents in hNPCs. 4-AP is traditionally used as a blocker of A-type potassium channels [24], [29]. In hNPCs 4-AP preferentially inhibited I_A_, but with less specificity. Since I_K_ was not completely blocked, IC_80_ values were used to block I_A_, but an inhibition of delayed-rectifying channels could not be excluded. Selective inhibition of K_v_1 delayed rectifier channels was obtained by DTX or MTX [31], [32]. Especially DTX sufficiently blocked K_v_1.1 and 1.6, which showed the highest expression levels among delayed-rectifying K_v_ channels in hNPCs. In hNPCs low doses of the classical Na^+^ channel blocker QND preferentially affected I_K_ (IC_50_ = 3 µM), while higher concentrations also inhibited I_A_ (IC_50_ = 43 µM). To obtain appropriate effects it was necessary to add 10 mM KCl to QND-treated cells due to its action as an open channel blocker [53], [54]. TEA, traditionally used as an inhibitor of DR potassium channels [24], [28], non-specifically blocked both current components and showed at best a slight preference in blocking I_K_. Hence, the biological effects of A-type channel inhibition were investigated in cell viability and proliferation assays using 4-AP and NH_4_Cl to preferentially and PTX to selectively block I_A_, while low doses of QND and DTX specifically inhibited I_K_. TEA acted as an unspecific K_v_ channel blocker in hNPCs.

Potassium channel function is assumed to be a key requirement for proper progenitor cell proliferation and also essential for functional neuronal differentiation [15]–[17], [66], [67]. In mature neurons K_v_ currents regulate neuronal excitability, while in undifferentiated neural progenitors they are speculated to be involved in cell proliferation [9]. By using the spider toxin PTX we were able to selectively block A-type channels and, thus, to investigate their specific contribution to cell viability and proliferation. In hNPCs a concentration-dependent reduction in cell viability and proliferation was observed after specific I_A_ inhibition with PTX. Less specific (4-AP, NH_4_Cl) as well as non-specific K_v_ antagonists (TEA, QND) showed similar toxicity. These results indicate that voltage-activated A-type currents generated predominantly by K_v_4.2 channels are likely to play a key role for proliferation and survival of hNPCs. This hypothesis is underlined by a downregulation of functional A-type channels with disrupting proliferation and inducing cell differentiation. Similar findings were obtained in adult neural progenitor cells, which showed an injury-induced increase in proliferation mediated by A-type K_v_4 channels [17]. Because of its fast activation and inactivation properties, I_A_ prevents mature neurons from responding to fast depolarizations [24], whereas in immature progenitor cells neuronal excitability is absent, but the occurrence of Ca^2+^ transients and their regulation by K^+^ channels has been described [67]. In this respect, the hyperpolarizing effect of K^+^ channels on the plasma membrane was thought to provide a driving force for the influx of Ca^2+^, which was believed to trigger cell proliferation [68], [69]. However, the exact mechanisms and tasks of I_A_ in proliferating neural progenitor cells remain to be fully elucidated. In contrast, the proliferation of oligodendrocyte progenitor cells is supposed to be controlled by the activity of several DR channels of the K_v_1 family [70], [71] suggesting different functions of K_v_ channels in neural and glial progenitors.

Furthermore, by using the snake toxin DTX we were able to selectively block I_K_. DTX did not cause accelerated cell death, but slightly increased proliferation of hNPCs. If we vice versa disrupted proliferation and induced differentiation, functional delayed-rectifier channels were upregulated. An increase in proliferation was also described in rat midbrain-derived NPCs after selective blockade of the DR channels K_v_1.3 and 3.1. Two explanations were described: First, a Ca^2+^ independent regulation via cell cycle mechanisms. Second, the mediation by a higher open probability of voltage-gated Ca^2+^ channels in response to the depolarizing effect caused by the K_v_ channel block and an increase of intracellular Ca^2+^ [8]. However, the fact that our data on differentiated hNPCs were obtained from a heterogeneous population of about 50% neuronal and 30% glial cells [10] allows no definitive conclusion about the role of delayed-rectifying potassium channels in the development of mature functional properties.

In summary, hNPCs generated K_v_ currents that consist to 90% of A-type currents predominantly produced by K_v_4.2 channels. Whereas delayed-rectifying currents mainly generated by K_v_1.1 and 1.6 were small. Inhibiting I_A_ function caused a dramatic decrease in proliferation and extensive cell death and, vice versa, disrupting proliferation reduced A-type current formation. These findings emphasize that even A-type potassium channels may play a key role in proliferation and survival of immature progenitor cells. On the other hand, the inhibition of I_K_ was less toxic and in case of DTX even increased progenitor cell proliferation. This is in line with the finding that non-proliferating, differentiating cells upregulated these channels.

## Supporting Information

Table S1 (0.04 MB DOC)

Table S2 (0.07 MB DOC)

Methods S1 (0.03 MB DOC)

Results S1 (0.03 MB DOC)

Results S2 (0.03 MB DOC)

Figure S1 Pharmacological inhibition of K_v_ currents in hNPCs by MTX. Delayed-rectifying (I_K_) K_v_ currents in proliferating hNPCs were inhibited by margatoxin (MTX), while A-type currents (I_A_) were not affected. (A): Peak amplitudes of I_A_ were measured during a depolarizing voltage step from 130 mV to 0 mV between 0 and 20 ms (inset). (B): I_K_ was determined between 280 and 300 ms of a 100 mV depolarization step following a −40 mV prepulse during the application of different antagonist concentrations (insets). (C): Both current values were normalized for the non-inhibited peak amplitudes. Dose-response relationships were fitted with the Hill equation and following parameters were obtained: IC_50_ = 180.7±46.9 nM, IC_80_ = 2.9 µM and a Hill coefficient of 0.5±0.1 (n = 4–8, mean±SD).
(5.11 MB TIF)

Figure S2 Cell cycle analysis after inhibition of voltage-gated potassium (K_v_) channels. Analysis of cell cycle phases in proliferating hNPCs was performed by means of flow cytometry using propidium iodide as an intercalating agent for DNA staining. (A): Cell cycle phases were determined after 72 h of K_v_ channel inhibition with 100 mM TEA, 2 mM 4-AP, 50 µM QND, 0.5 µM DT\and 0.1 µM MTX and their distribution was calculated by dividing through the total cell number. (B): Cell cycle rates were normalized to controls without addition of an inhibitor. The application of TEA and 4-AP increased cell death about 7 times, while G1/G0, G2/M and S phase were decreased compared to control. QND, DTX and MTX were less toxic (n = 10,000, 4 experiments; one-way ANOVA, followed by Tukey's post-hoc test, *p<0.05, ***p<0.001).
(8.73 MB TIF)
