# Supplementary material for: Characterization of Voltage-Gated Potassium Channels in Human Neural Progenitor Cells
Source: PLoS One. 2009 Jul 8;4(7):e6168. doi: 10.1371/journal.pone.0006168 (PMC2702754; doi:10.1371/journal.pone.0006168)
Supplement: Table S1 — (0.04 MB DOC) [file pone.0006168.s001.doc]

**Table S1.** Overview of voltage-gated potassium (K_v_) channel subtypes (modified after Alexander et al. [58]).

| Subfamily | K_v_1.x | K_v_2.x | K_v_3.x | K_v_4.x |
| --- | --- | --- | --- | --- |
|  | **Shaker** | **Shab** | **Shal** | **Shaw** |
| Subtype | K_v_1.1 - 1.7 | K_v_2.1 - 2.2 | K_v_3.1 - 3.4 | K_v_4.1 - 4.3 |
| Inhibitors | TEA (1.1) | TEA | TEA | PTX (4.2,4.3) |
|  | TEA (1.3,1.6) |  | 4-AP (3.1,3.2) |  |
|  | 4-AP (1.4) |  |  |  |
|  | α-DTX (1.1,1.2,1.6) |  |  |  |
|  | rMTX (1.1,1.2,1.3) |  |  |  |
| Functional  characteristics | I_K_ (1.1-1.3,1.5-1.7)  I_A_ (1.4) | I_K_ (2.1) | I_K_ (3.1,3.2)  I_A_ (3.3,3.4) | I_A_ |
