# Supplementary material for: Characterization of Voltage-Gated Potassium Channels in Human Neural Progenitor Cells
Source: PLoS One. 2009 Jul 8;4(7):e6168. doi: 10.1371/journal.pone.0006168 (PMC2702754; doi:10.1371/journal.pone.0006168)
Supplement: Table S2 — (0.07 MB DOC) [file pone.0006168.s002.doc]

**Table S2.** Specific primers for qRT-PCR of voltage-gated potassium (K_v_) channel subtypes.

| **K_v_-Subtype** | **Gene** | **Product**  **(bp)** | **T_M_**  **(°C)** | **Primer** | **Sequence** |
| --- | --- | --- | --- | --- | --- |
| K_v_1.1 | KCNA1 | 143 | 61.4 | fw | 5’-CTCTAAGGGCCTCCAGATCC-3’ |
|  |  |  | 59.4 | rev | 5’-AGTGCGACTCAGCTTCTTCC-3’ |
| K_v_1.2 | KCNA2 | 99 | 57.3 | fw | 5’-TGGTGCATCTCAGATTCCTG-3’ |
|  |  |  | 51.8 | rev | 5’-TCTGAGAGCTGGAGAGACAGC-3’ |
| K_v_1.3 | KCNA3 | 115 | 56.7 | fw | 5’-CGCGTGGTCATCAACATCT-3’ |
|  |  |  | 58.8 | rev | 5’-GCGGGTCGAAGTACCTCAT-3’ |
| K_v_1.4 | KCNA4 | 132 | 55.3 | fw | 5’-CAGCCGGTGGATTTTCTTTA-3’ |
|  |  |  | 59.4 | rev | 5’-GCAAAACCAGCTCTGAGGTC-3’ |
| K_v_1.5 | KCNA5 | 123 | 61.4 | fw | 5’-ACTGGTGGCAGTGGAGTAGG-3’ |
|  |  |  | 59.4 | rev | 5’-GGGAGGAAAGGAGTGAAAGG-3’ |
| K_v_1.6 | KCNA6 | 124 | 57.3 | fw | 5’-CTGAGATGCGGGATATTCGT-3’ |
|  |  |  | 59.4 | rev | 5’-GGTCCAGCTGCTGTCAGAAT-3’ |
| K_v_1.7 | KCNA7 | 96 | 57.3 | fw | 5’-CCTTCAATGACCCGTTCTTC-3’ |
|  |  |  | 59.4 | rev | 5’-TAGCCTTGCTTGGACAGACC-3’ |
| K_v_2.1 | KCNB1 | 128 | 57.3 | fw | 5’-ACTGGAGAAGCCCAATTCCT-3’ |
|  |  |  | 59.4 | rev | 5’-CGAACTCATCGAGGCTCTGT-3’ |
| K_v_2.2 | KCNB2 | 97 | 57.3 | fw | 5’-TTTTAAGGACCCTGGCTCTG-3’ |
|  |  |  | 57.3 | rev | 5’-GGGAAAGTGTCGACCTTGAA-3’ |
| K_v_3.1 | KCNC1 | 102 | 59.4 | fw | 5’-CCTTCGCTTCCCTCTTCTTC-3’ |
|  |  |  | 59.4 | rev | 5’-CGTTCTCGATCTCCGTCTTG-3’ |
| K_v_3.2 | KCNC2 | 108 | 57.3 | fw | 5’-GTGACACATGTCTGGGCAAA-3’ |
|  |  |  | 59.4 | rev | 5’-GCCTTTCTGGGGGTGATAGT-3’ |
| K_v_3.3 | KCNC3 | 108 | 56.0 | fw | 5’-AAGCTTCTTGCCCGACCT-3’ |
|  |  |  | 59.4 | rev | 5’-AAAGTCTCGCGAGGTCTCAG-3’ |
| K_v_3.4 | KCNC4 | 121 | 61.4 | fw | 5’-GCTCAGCACTGGGGACTATG-3’ |
|  |  |  | 57.3 | rev | 5’-GTTGGTGCCGCTTTAAGAGA-3’ |
| K_v_4.1 | KCND1 | 142 | 57.3 | fw | 5’-AGGGCACAAACAAGACCAAC-3’ |
|  |  |  | 59.4 | rev | 5’-CCACTGAGTGAGCAGATGGA-3’ |
| K_v_4.2 | KCND2 | 139 | 55.3 | fw | 5’-ACAAACGAAGGGCACAAAAG-3’ |
|  |  |  | 57.3 | rev | 5’-AAAGCCTGCTCATCCTCTGA-3’ |
| K_v_4.3 | KCND3 | 121 | 59.4 | fw | 5’-GGCAAGACCACCTCACTCAT-3’ |
|  |  |  | 57.3 | rev | 5’-TGATGGTGGAGGTTCGTACA-3’ |
| RPL22 | RPL22 | 116 | 51.9 | fw | 5’-CACGAAGGAGGAGTGACTGG-3’ |
|  |  |  | 51.6 | rev | 5’-TGTGGCACACCACTGACATT-3’ |
