# Supplementary material for: Characterization of Voltage-Gated Potassium Channels in Human Neural Progenitor Cells
Source: PLoS One. 2009 Jul 8;4(7):e6168. doi: 10.1371/journal.pone.0006168 (PMC2702754; doi:10.1371/journal.pone.0006168)
Supplement: Methods S1 — (0.03 MB DOC) [file pone.0006168.s003.doc]

# Methods S1

**Flow cytometry for apoptosis and cell cycle analysis**

Cell cycle analysis was performed by means of flow cytometry using propidium iodide to estimate DNA content. Therefore, hNPCs were plated on 12‑well PLO / FN-precoated culture plates in a density of 100,000 cells / well. Following 5 days of proliferation K_v_ antagonists were applied for 72 h: TEA (100 mM), 4-AP (2 mM), QND (50 µM), DTX (1 µM) and MTX (100 nM) in PM. Untreated (control) and treated hNPCs were collected via accutase detachment (PAA Laboratories GmbH, Pasching, Austria) and centrifugation, and prepared for cell cycle analysis by lysing cells in 300 µl hypotonic lysis buffer (0.1 % sodium citrate, 0.1 % Triton X‑100, 50 µg/ml propidium iodide). After at least 30 min of incubation at 4°C, cells were analyzed by flow cytometry on a FACScan (Becton Dickinson, Heidelberg, Germany), using 488 nm excitation, gating out doublets and clumps using pulse processing, and collecting fluorescence above 620 nm according to the method of Nicoletti et al. (1991) [72]. A minimum of 10,000 events was collected for each experimental sample. Histograms of DNA content were acquired using CellQuest software (Becton Dickinson, Heidelberg, Germany). The number of nuclei present in each peak of the histogram (sub‑G1, G1/G0, S, G2/M) was analyzed by measuring the peak area. For data analysis and correction of background, noise histograms were processed with ModFit LT software (Verity, Turramurra, NSW, Australia) [37].

# References S1

72. Nicoletti I, Migliorati G, Pagliacci MC, Grignani F, Riccardi C (1991) A rapid and simple method for measuring thymocyte apoptosis by propidium iodide staining and flow cytometry. J Immunol Methods 139: 271-279.
