# Supplementary material for: Characterization of Voltage-Gated Potassium Channels in Human Neural Progenitor Cells
Source: PLoS One. 2009 Jul 8;4(7):e6168. doi: 10.1371/journal.pone.0006168 (PMC2702754; doi:10.1371/journal.pone.0006168)
Supplement: Results S1 — (0.03 MB DOC) [file pone.0006168.s004.doc]

# Results S1

**Pharmacological inhibition of K_v_ currents in hNPCs by MTX**

To selectively inhibit I_K_ the neurotoxin margatoxin (MTX) was applied in addition to α‑dendrotoxin (DTX). Both are considered to acts as antagonists on K_v_1 channels [32]. MTX specifically inhibited I_K_ in hNPCs, while it was ineffective in blocking I_A_ (Fig. S1). MTX showed a similar concentration dependency (slope 0.5) and IC_50_ value (180.7 nM) compared to DTX (IC_50_ = 163.9 nM, slope 0.7; Fig. S1). Because the channel transcripts K_v_1.2 and 1.3 showed low expression levels, it predominantly inhibited K_v_1.1, while DTX additionally blocked K_v_1.6 (Fig.3, Tab. S1).
