# Supplementary material for: Characterization of Voltage-Gated Potassium Channels in Human Neural Progenitor Cells
Source: PLoS One. 2009 Jul 8;4(7):e6168. doi: 10.1371/journal.pone.0006168 (PMC2702754; doi:10.1371/journal.pone.0006168)
Supplement: Results S2 — (0.03 MB DOC) [file pone.0006168.s005.doc]

# Results S2

**Biological effects of K_v_ channel inhibiton on cell cycle of hNPCs**

To substantiate the results obtained in viability and proliferation tests we analyzed the effects of K_v_ channel inhibition on cell cycle of hNPCs. Therefore, we performed flow cytometry using the DNA-intercalating agent propidium iodide to determine DNA content and cell cycle phases. Prior to the flow cytometric analysis, the K_v_ channel inhibitors were applied for 3 days (Fig. S2 A). Apoptotic cells were identified by a subdiploid peak (sub-G1). Survival of untreated cells was > 94 %. Treatment with TEA and 4-AP increased cell death 7‑fold compared to control, while QND, DTX and MTX caused no significant increase (less than 2‑fold). Furthermore, TEA and 4‑AP significantly influenced the cell cycle by decreasing the percentage of cells in G0/G1, G2/M and S-phase (Fig. S2 B, C). In comparison to 75 % of untreated cells being non-dividing or growing in G0/G1-phase, the application of TEA and 4-AP decreased this cell cycle phase to 55 %, while QND, DTX and MTX had no considerable effect on growth of hNPCs. G2/M-phase (5 % in control cells) was reduced by TEA and QND (3 %) and almost completely inhibited by 4‑AP, which consequently impaired cell divisions. MTX and DTX caused no significant decrease (4 ‑ 5 %). S‑phase (15 % in control cells) was affected primarily by TEA and 4-AP, which reduced DNA synthesis in hNPCs to 6 %, while QND, DTX and MTX were less effective (13 ‑ 14 %; Fig. 5 C). Thus, the intense cell death of hNPCs in response to 4-AP or TEA was caused by an arrest in cell cycle and especially in case of 4-AP by strongly impaired cell divisions. In comparison, blocking I_K_ by QND also inhibited cell divisions, but however, there was no significant effect on cell survival in response to QND, DTX or MTX.
