# Supplementary figures and images for: Characterization of Voltage-Gated Potassium Channels in Human Neural Progenitor Cells
Source: PLoS One. 2009 Jul 8;4(7):e6168. doi: 10.1371/journal.pone.0006168 (PMC2702754; doi:10.1371/journal.pone.0006168)

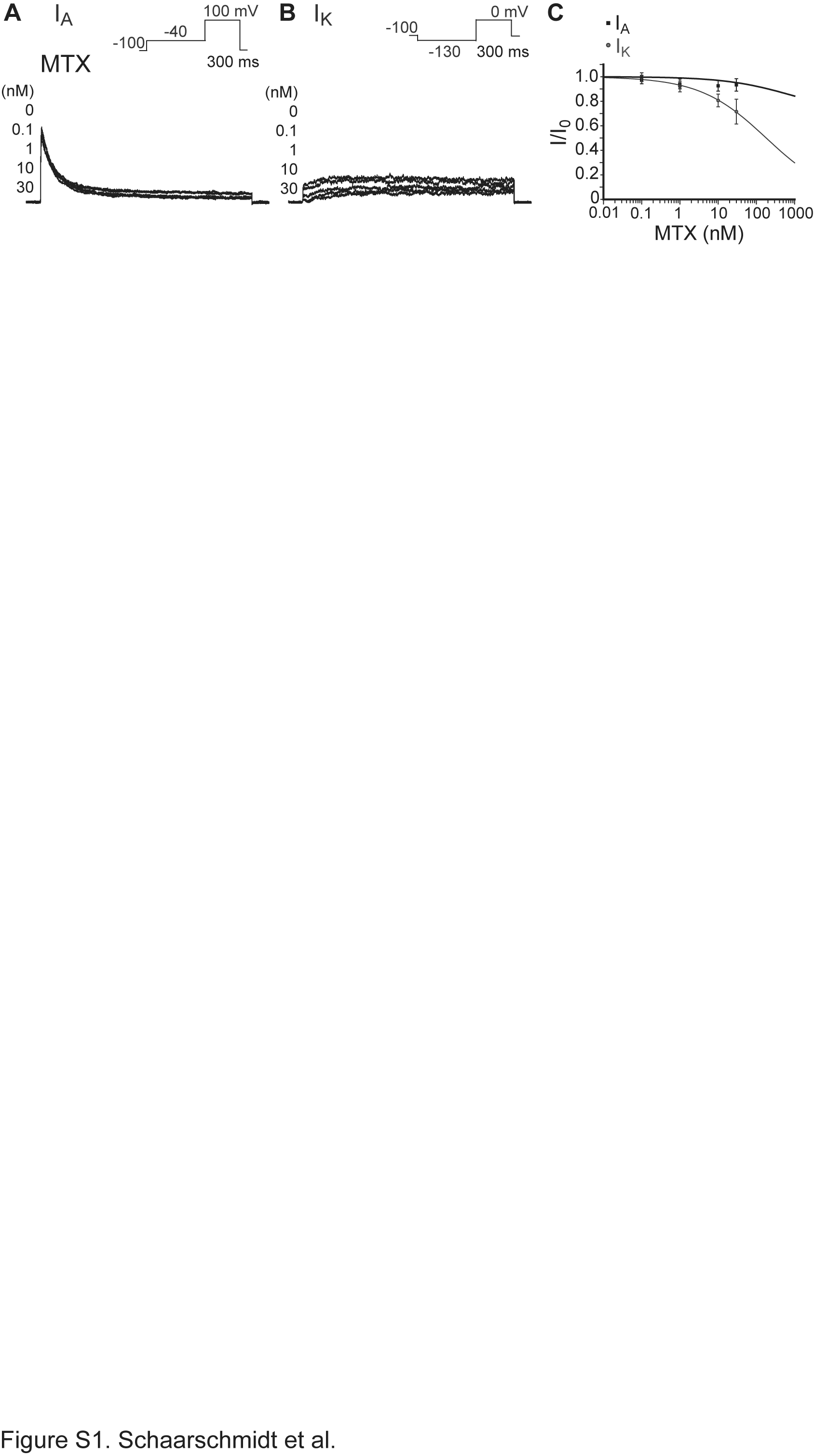

Supplement: Figure S1 — Pharmacological inhibition of Kv currents in hNPCs by MTX. Delayed-rectifying (IK) Kv currents in proliferating hNPCs were inhibited by margatoxin (MTX), while A-type currents (IA) were not affected. (A): Peak amplitudes of IA were measured during a depolarizing voltage step from 130 mV to 0 mV between 0 and 20 ms (inset). (B): IK was determined between 280 and 300 ms of a 100 mV depolarization step following a −40 mV prepulse during the application of different antagonist concentrations (insets). (C): Both current values were normalized for the non-inhibited peak amplitudes. Dose-response relationships were fitted with the Hill equation and following parameters were obtained: IC50 = 180.7±46.9 nM, IC80 = 2.9 µM and a Hill coefficient of 0.5±0.1 (n = 4–8, mean±SD). (5.11 MB TIF) [file pone.0006168.s006.tif]

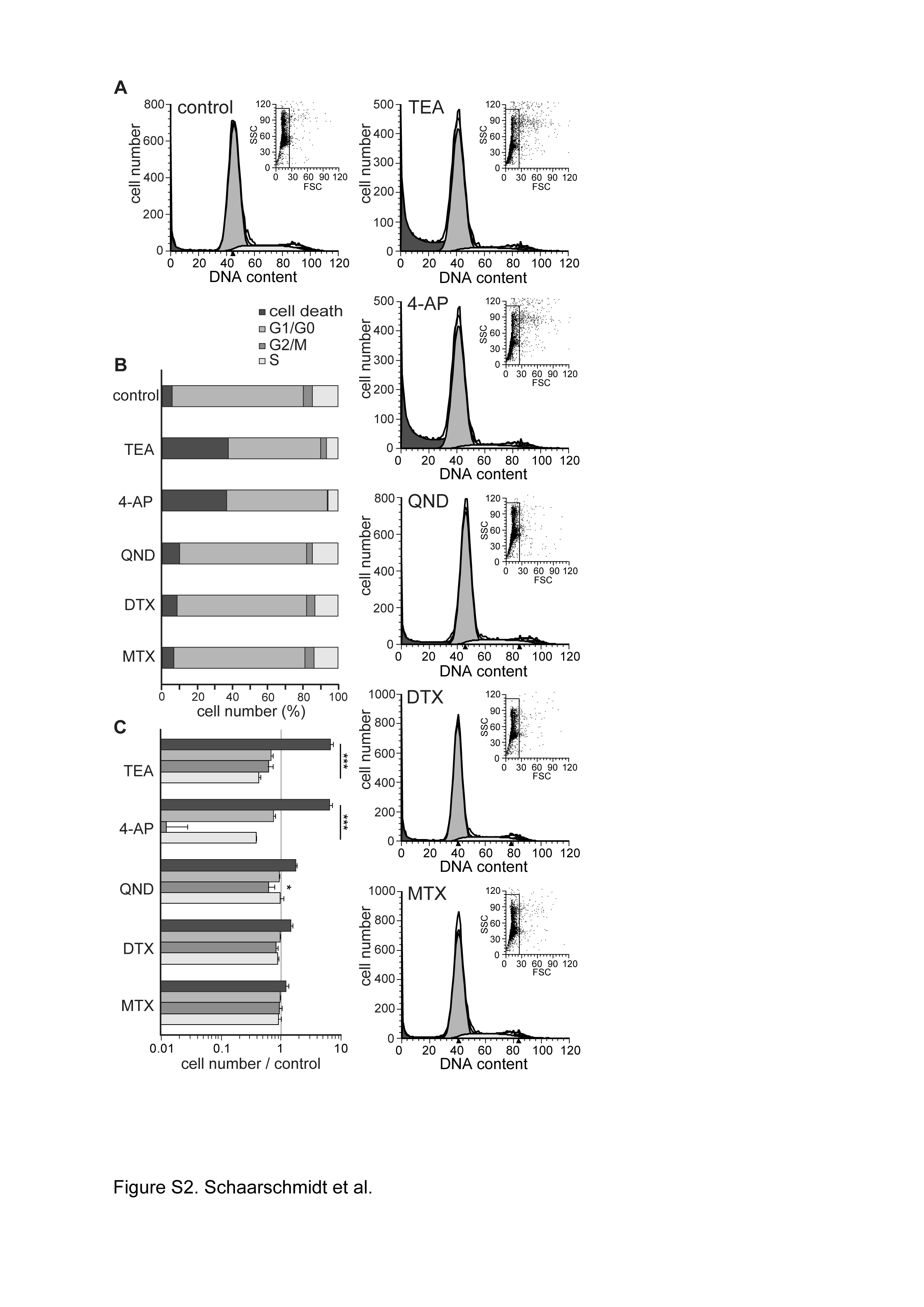

Supplement: Figure S2 — Cell cycle analysis after inhibition of voltage-gated potassium (Kv) channels. Analysis of cell cycle phases in proliferating hNPCs was performed by means of flow cytometry using propidium iodide as an intercalating agent for DNA staining. (A): Cell cycle phases were determined after 72 h of Kv channel inhibition with 100 mM TEA, 2 mM 4-AP, 50 µM QND, 0.5 µM DT\and 0.1 µM MTX and their distribution was calculated by dividing through the total cell number. (B): Cell cycle rates were normalized to controls without addition of an inhibitor. The application of TEA and 4-AP increased cell death about 7 times, while G1/G0, G2/M and S phase were decreased compared to control. QND, DTX and MTX were less toxic (n = 10,000, 4 experiments; one-way ANOVA, followed by Tukey's post-hoc test, *p<0.05, ***p<0.001). (8.73 MB TIF) [file pone.0006168.s007.tif]
